# Breast Cancer in Women Aged ≤35 Years: A Single-Center Retrospective Comparative Analysis by Age Subgroup

**DOI:** 10.3390/diagnostics16162510

**Published:** 2026-08-09

**Authors:** Ebru Dusunceli Atman, Sena Bozer Uludag, Caglar Uzun, Gizem Agaran, Zeynep Eskalen

**Affiliations:** 1 Department of Radiology, School of Medicine, Ankara University, Altındag, 06230 Ankara, Türkiye; ebrumd2001@yahoo.com (E.D.A.); gizemagaran@gmail.com (G.A.); zeynep_cotaoglu@hotmail.com (Z.E.); 2 Department of Radiology, Tosya State Hospital, Tosya, 37300 Kastamonu, Türkiye; senabozer@gmail.com

**Keywords:** breast cancer, magnetic resonance imaging, mammography, ultrasound, young women

## Abstract

**Background/Objectives**: The prognostic significance of young age in breast cancer (BC) remains controversial, with varying findings across studies. This study aimed to evaluate the imaging characteristics, pathological features, and survival outcomes of BC in young patients aged ≤ 35 years by dividing them into two groups and to compare outcomes between these subgroups to determine whether very young age alone is associated with worse prognosis. **Methods**: This retrospective study included patients aged ≤ 35 years with malignant breast lesions who underwent image-guided interventions at a single center between January 2011 and December 2024. Patients were divided into two groups: Group 1 (≤30 years) and Group 2 (31–35 years). Demographic, imaging, and pathological data, as well as survival outcomes, were analyzed. **Results**: A total of 34 patients (Group 1: *n* = 14, Group 2: *n* = 20) with malignant breast lesions underwent 40 image-guided procedures. No significant differences were observed between groups in imaging features, pathological characteristics, or pTNM stage distribution. Median follow-up was 68 months. One-year overall survival (OS) was 100% in both cohorts; 5-year OS rates were 100% and 92.3%, respectively, with no statistically significant difference (*p* = 0.784). Median event-free survival (EFS) was 60.5 months, also without a significant difference between groups (*p* = 0.897). None of the clinicopathological factors assessed were significantly associated with OS in this exploratory analysis. **Conclusions**: In women ≤ 35 years, tumor characteristics and patient outcomes were similar between those aged ≤ 30 and 31–35 years. OS and EFS were favorable in both groups, indicating that within the ≤35-year population studied, being ≤30 years old was not associated with worse outcomes than being 31–35 years old. Although advanced stage disease was more frequently observed in the younger group, this difference was not statistically significant.

## 1. Introduction

Breast cancer (BC) is the most common malignancy among women and the leading cause of cancer-related death in females worldwide [1,2]. BC is rare in women under 35, but it remains the leading cause of cancer-related death in this age group [3,4,5]. Similar to the overall rise in cancer incidence, BC demonstrates an age-dependent increase. However, a gradual rise in BC incidence has also been documented among the young population. While the incidence of BC in women under 40 years of age accounts for approximately 4% in Western countries, this proportion is notably higher in East Asian nations, with China reporting a rate of 16.4% in 2017 [3,6,7,8].

It is still unclear whether being diagnosed with primary BC at a young age leads to worse outcomes. Some studies have indicated poorer clinical outcomes and higher risks of recurrence or death in younger patients compared with older patients, while others have reported similar or even more favorable outcomes [3,6,9,10,11,12,13]. These inconsistencies may be explained by small study cohorts, heterogeneous patient selection, differences in ethnic characteristics, and varying definitions of “young age.” Indeed, there is no consensus on the age cut-off, with various thresholds applied across studies. Increasing evidence suggests that tumor biology plays a central role, as BC in younger women is more frequently of higher grade, demonstrates greater proliferative activity and vascular invasion, and shows lower expression of estrogen and progesterone receptors (ER, PR) compared with that in older women [3,6,7,8,14,15,16]. In young women, the lack of routine BC screening protocols—aside from high-risk individuals—coupled with the inherently limited mammographic sensitivity in the setting of dense breast tissue, pregnancy, and lactation, results in a disproportionately high rate of advanced disease at initial presentation [3,8,17,18].

The present study was designed to directly compare imaging characteristics, pathological features, and survival outcomes between two age subgroups of young BC patients who were managed with the same treatment strategies at a single institution, rather than comparing them with postmenopausal counterparts. Nevertheless, as the mean age at BC diagnosis in the Middle East, East Asia and Africa is approximately one decade lower than that observed in Western populations [19,20], a cut-off age of 30 years was deemed more appropriate and adopted accordingly in this study. Young patients were therefore stratified into two groups for comparative analysis: those aged 30 years or younger, and those between 31 and 35 years of age. By focusing on these subgroups, we aimed to investigate whether clinically relevant differences exist within this already young patient population, and whether very young age alone constitutes an independent adverse prognostic factor, based on a 14-year single-center experience.

## 2. Materials and Methods

This retrospective study was approved by the Institutional Human Research Ethics Committee and patient data were anonymized before analysis.

**Figure 1:**
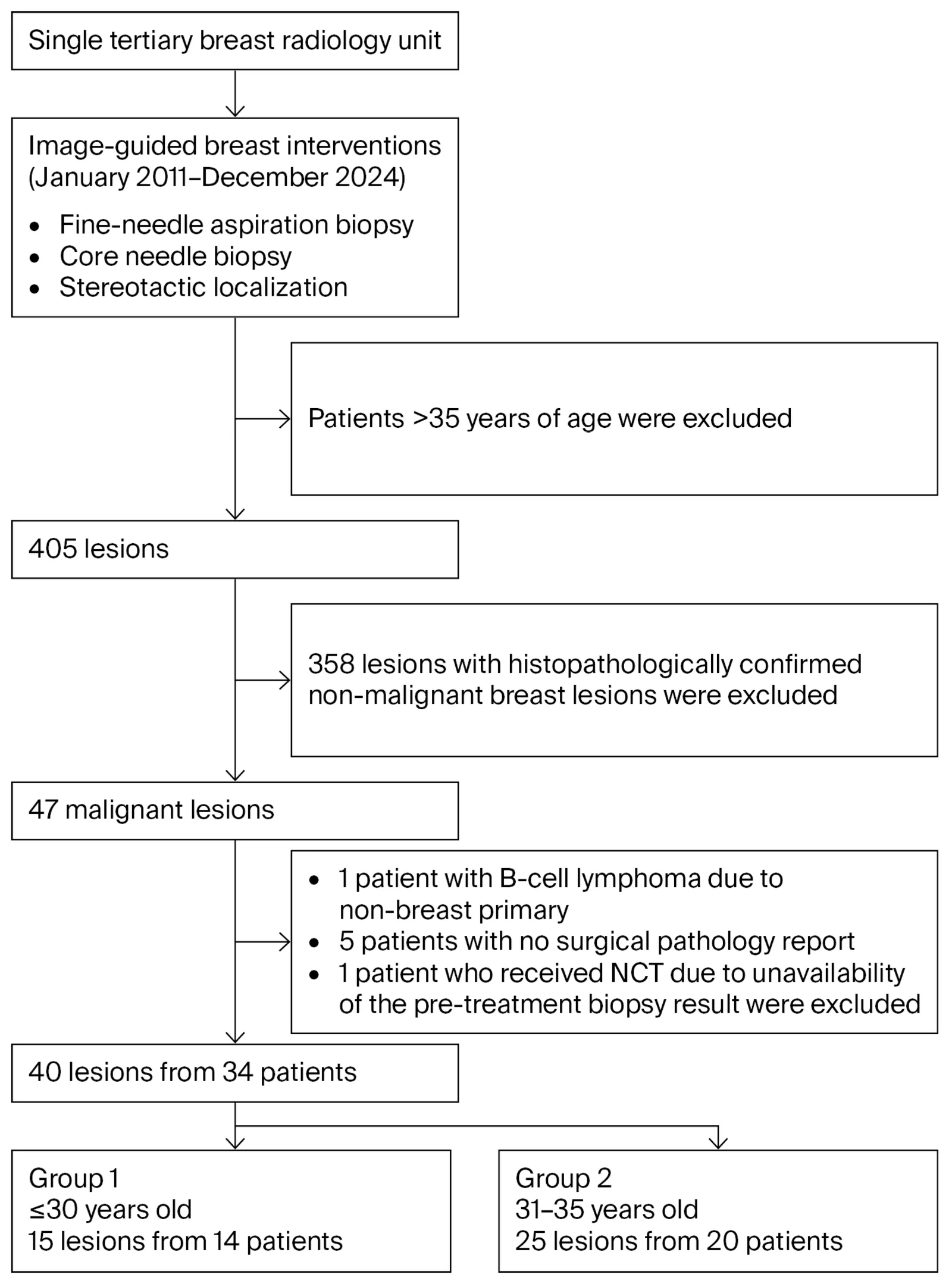
Flowchart illustrating patient selection and the exclusion process.

Our study was conducted at a single tertiary referral center over a 14-year period and included patients aged 35 years or younger with malignant breast lesions. All patients who underwent image-guided breast interventions—including fine-needle aspiration biopsy (FNAB), core needle biopsy, and/or stereotactic-guided excisional biopsy—between January 2011 and December 2024 were retrospectively reviewed. Five patients who had surgery at other institutions, one patient who had received neoadjuvant chemotherapy (NCT) at another center before stereotactic-guided excisional biopsy, and one patient with a history of systemic B-cell lymphoma were excluded. Patients were grouped by age: Group 1 (≤30 years) and Group 2 (31–35 years). An overview of the patient inclusion and exclusion criteria is provided in the flowchart (Figure 1).

Patient demographics, clinical characteristics (family history, pregnancy, lactation period), imaging findings—ultrasound (US), mammography, and magnetic resonance imaging (MRI)—pathology results, and survival outcomes were retrieved from the electronic records. Lesion laterality, locations, and distribution were documented. Pathological tumor data included size, histological type, grade, and immunohistochemical results. Surgical specimens were used as the reference standard; however, for patients who had received NCT, pre-treatment biopsy findings were considered instead. The 8th edition of the Tumor, Nodes and Metastasis (TNM) system was used to determine tumor stage [17]. Pathological TNM (pTNM) staging was determined based on surgical specimens. Patients who underwent NCT were excluded from pTNM analysis to prevent treatment-related alterations in staging. Immunohistochemical results—ER, PR, and human epidermal growth factor receptor 2 (HER2) status, and Ki-67 index—were noted. A threshold of 10% was used to define positive expression for ER and PR [21]. In cases with equivocal HER2 immunohistochemistry results, fluorescence in situ hybridization (FISH) was performed to clarify HER2 status. Tumoral subtypes were categorized as luminal A (ER+ and/or PR+, Ki-67 < 14% and HER2−); luminal B (ER+ and/or PR+, Ki-67 ≥ 14% and/or HER2+); HER2− enriched (ER−, PR−, and HER2+) and triple negative (ER−, PR−, and HER2−) [21,22].

All patients underwent US examination (LOGIQ S7 Expert, LOGIQ S8, LOGIQ P9, GE Healthcare, Milwaukee, WI, USA) prior to the interventional procedure. When clinically indicated, additional imaging such as mammography (Siemens Mammomat Inspiration (Erlangen, Germany) and Hologic Selenia Dimensions (Hologic, Inc., Bedford, MA, USA), MRI (3T, Siemens Magnetom Verio Syngo MR B17, Erlangen, Germany) and positron emission tomography was also performed. All examinations were retrospectively reviewed and evaluated in accordance with the American College of Radiology Breast Imaging Reporting and Data System (ACR BI-RADS) lexicon [23]. The images were analyzed by two radiologists with 23 and 4 years of experience in breast imaging, and any discrepancies were resolved by consensus.

All patients had a minimum follow-up of six months. Survival analysis was based on two outcomes: overall survival (OS), defined as the time from diagnosis to death from any cause, with patients alive at last follow-up censored at that date; and event-free survival (EFS) refers to the time from diagnosis to the first occurrence of local recurrence, distant metastasis, contralateral BC, or death from any cause, whichever came first, with patients free of an event censored at last follow-up. EFS analyses were performed for patients with available follow-up data. Recurrence was defined as either local recurrence and/or the development of distant metastasis, confirmed histopathologically or based on imaging findings from modalities such as PET-CT. Patients with no follow-up visit within the last year were excluded from the survival analyses.

### Statistical Analysis

Statistical analyses were performed using SPSS 17.0 (Chicago, IL, USA). Categorical variables were expressed as frequencies (%) and compared via chi-square or Fisher’s exact tests. Continuous variables were presented as means ± standard deviations (SD) or medians with interquartile ranges (IQR) and analyzed using t-tests or Mann–Whitney U tests. Effect sizes and measurement precision were systematically evaluated for all comparisons. Cramer’s V (*V*) was computed for multi-category contingency tables, whereas the Phi coefficient (*φ*) alongside Odds Ratios (ORs) with their corresponding 95% Confidence Intervals (95% CIs) were reported for binary categorical outcomes. Effect size magnitudes were interpreted according to standard thresholds: 0.10 to <0.30 as a small effect, 0.30 to <0.50 as a medium effect, and ≥0.50 as a large effect. Survival rates (1- and 5-year EFS/OS) were estimated using Kaplan–Meier analysis and compared with log-rank tests. Univariate Cox regression evaluated factors affecting OS. The proportional hazards assumption was formally verified using scaled Schoenfeld residuals and visual inspection of log-log survival curves. However, the limited number of events precluded multivariable modeling. A *p*-value < 0.05 was considered significant. Given the small subgroup sizes, non-significant comparisons should be interpreted with caution and do not establish equivalence between groups; this study should be regarded as exploratory and hypothesis-generating rather than confirmatory, with a non-trivial risk of Type II error.

## 3. Results

### 3.1. Study Population

Given the small subgroup sizes (Group 1: *n* = 14; Group 2: *n* = 20, with 11 and 19 patients, respectively, available for survival analysis), the statistical power to detect true between-group differences was limited; non-significant *p*-values reported throughout should therefore be interpreted with this constraint in mind, rather than as evidence of true similarity between groups.

In total, 40 image-guided procedures with histopathological diagnosis were performed in 34 patients (Group 1: *n* = 14; Group 2: *n* = 20); 28 had one lesion, while six had two lesions sampled in the same breast. Additional satellite lesions observed on imaging were not included unless biopsied. All analyses were conducted on a per-patient basis, except for lesion-based parameters, including imaging characteristics and pathological tumor features, which were analyzed at the lesion level (*n* = 40). Clinical variables (age, family history, pregnancy status) and survival outcomes (OS and EFS) were evaluated on a per-patient basis (*n* = 34).

The mean age was 30.5 ± 3.7 years overall, with 26.9 ± 3.0 years (range: 19–30) in Group 1 and 32.9 ± 1.2 years (range: 31–35) in Group 2. Two patients were diagnosed during lactation and one during pregnancy. Family history, defined as having a first-degree relative with a known diagnosis of BC, was present in six patients (17.6%; Group 1: 14.3%, Group 2: 20%), with no significant difference between groups. Genetic testing in eight patients revealed no mutations in five cases, while three patients carried BRCA1, MSH6, and CHEK2/POLD1 mutations.

### 3.2. Radiologic Assessment

**Figure 2:**
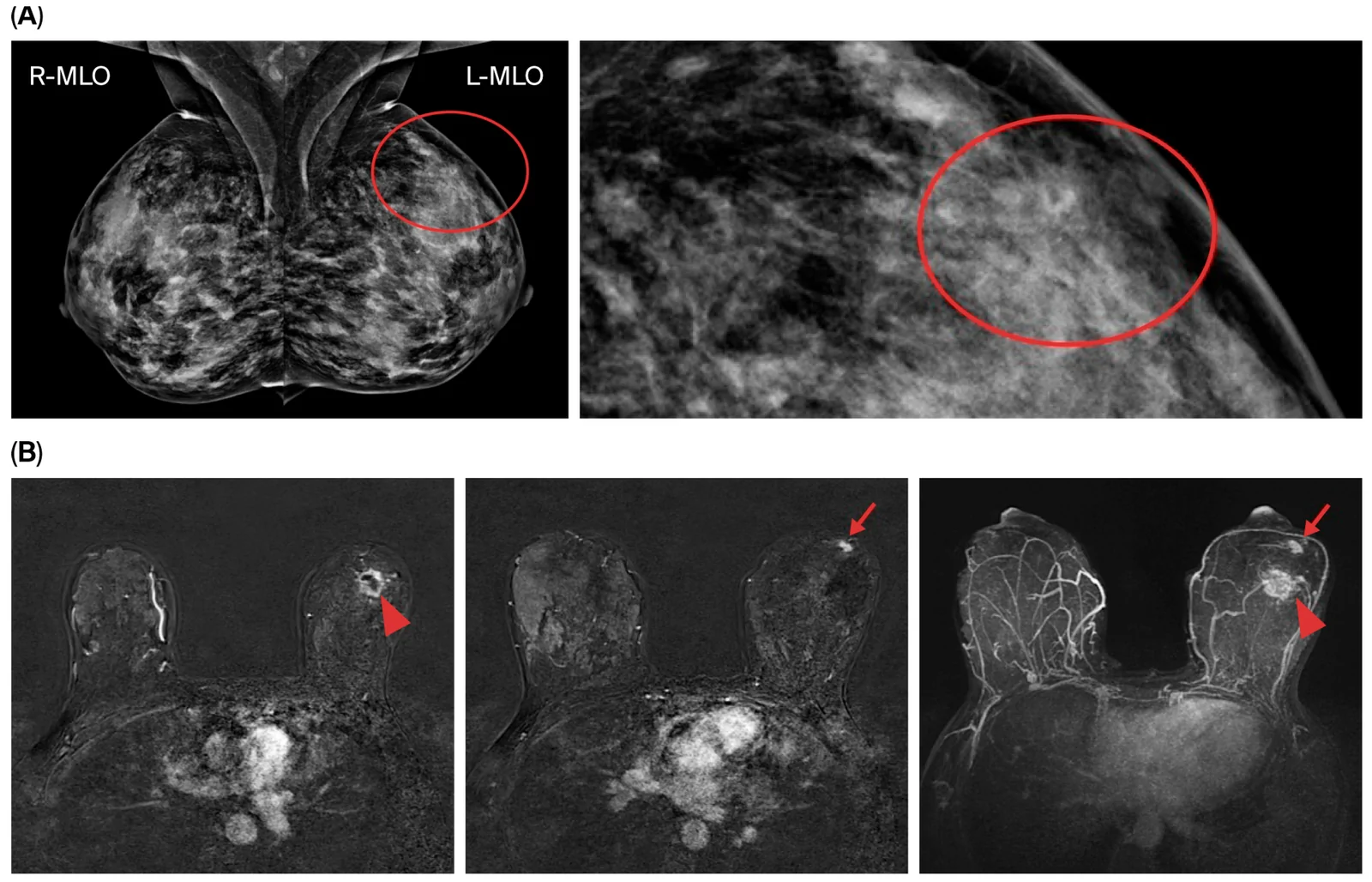
A 31-year-old female with multifocal left breast cancer diagnosed during the lactation period. (**A**) Bilateral MLO mammograms demonstrate extremely dense breasts (BI-RADS density type D), markedly limiting lesion visibility. Although no discrete mass is identified, grouped microcalcifications are evident in the upper half of the left breast (circle). (**B**) Axial post-contrast subtracted T1-weighted images were obtained at two different levels, along with a MIP image. The subtracted images show a spiculated mass with peripheral enhancement and central necrosis in the upper outer quadrant of the left breast, lateral to the midline (arrowhead). The lesion exhibits type 3 kinetic enhancement. An additional enhancing focus is present (arrow), immediately caudal and anterior to the primary lesion, consistent with multifocal disease. The MIP image displays both lesions simultaneously, confirming the multifocal nature of the cancer. MLO: mediolateral oblique.

**Figure 3:**
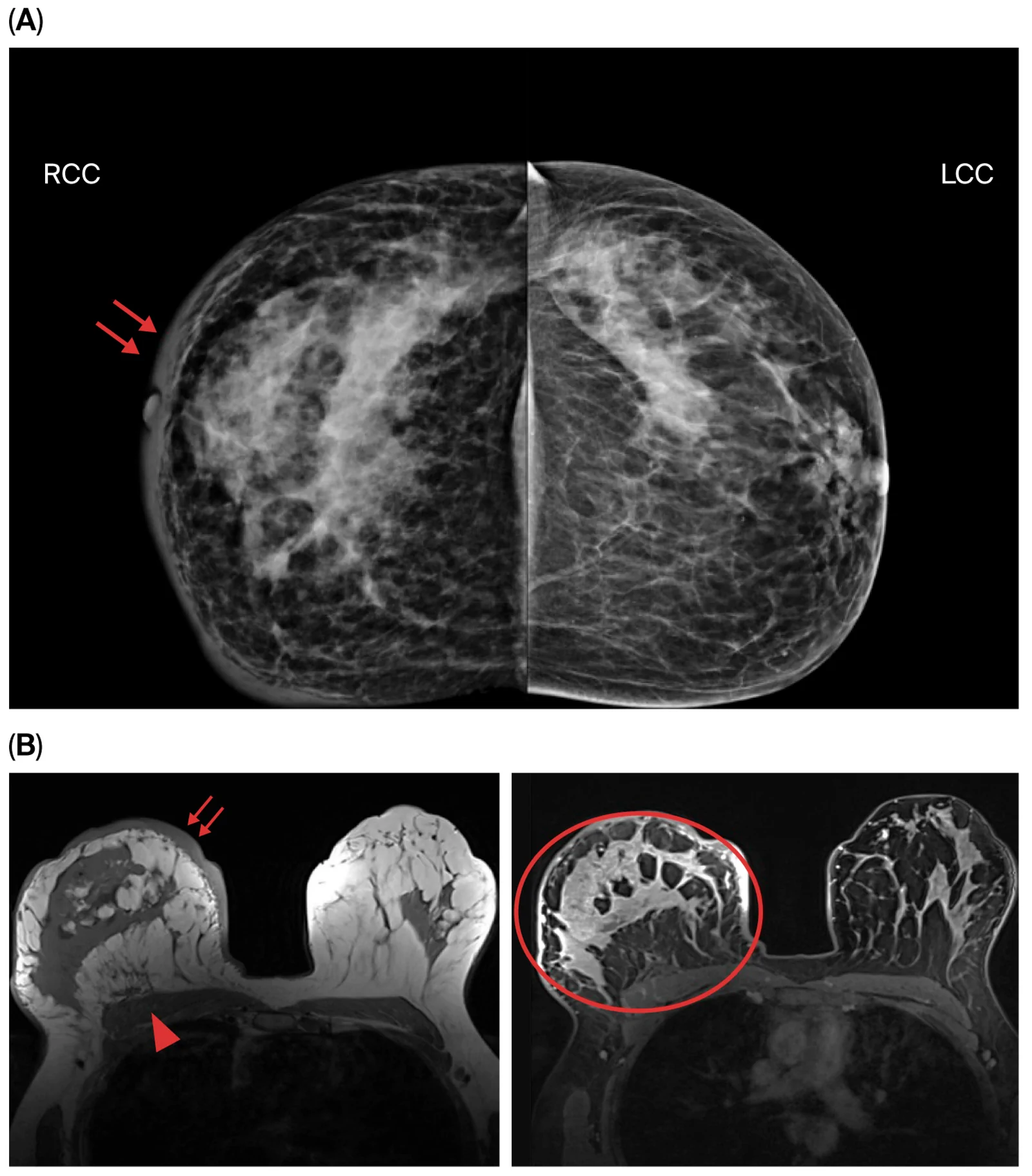
A 34-year-old woman with inflammatory breast cancer. (**A**) Bilateral CC mammograms. The right CC view demonstrates diffuse skin thickening (arrows) and increased breast density. The left breast appears normal. (**B**) Axial T2-weighted image and axial contrast-enhanced T1-weighted image demonstrate marked enlargement of the right breast with diffuse skin thickening (arrows), a large isointense infiltrative mass showing diffuse non-mass-like enhancement with a type 3 kinetic curve (circle), and retraction of the right pectoralis muscle (arrowhead). RCC: right craniocaudal, LCC: left craniocaudal.

US was obtained for all patients, whereas mammograms and MRIs were available for 14 and 10 patients, respectively. BI-RADS categorization was applied for imaging modalities. Detailed radiologic findings, including ultrasonographic, mammographic, and MRI features for both groups, are provided in Table 1 (detailed imaging findings and pathological tumor data are provided in the Supplementary File). All tumors were unilateral, including unifocal (64.7%), multifocal (32.4%; Figure 2), and multicentric (2.9%) diseases, with no significant differences in laterality (*p* = 0.291, *φ* = 0.214; OR = 2.500, 95% CI: 0.569–10.985). Lesions presented as masses in 80% (*n* = 32) and non-masses in 20% (*n* = 8) of cases. Mass presentation predominated across both cohorts (73.3% vs. 84.0%). Although non-mass presentation was numerically higher in Group 1 (26.7% vs. 16.0%), this difference was not statistically significant, reflecting a small effect magnitude (*p* = 0.435, *φ* = 0.128; OR = 1.909, 95%CI: 0.395–9.222). Masses were predominantly irregular (87.5%), microlobulated (40.6%), and hypoechoic (78.1%). Calcifications were present in 20 lesions (50%), most commonly within the mass (43.7%). Associated US features included architectural distortion (20%), duct changes (15%), skin thickening (7.5%) (Figure 3), skin retraction (2.5%), and edema (2.5%). None of the US imaging features demonstrated a statistically significant difference between the two age groups. Although the distribution of BI-RADS categories showed no statistically significant difference (*p* = 0.322), a small-to-moderate effect magnitude was observed (*φ* = 0.181). Specifically, lesions in Group 1 exhibited a higher propensity for BI-RADS 5 assignment than those in Group 2 (66.6% vs. 48.0%, respectively; OR = 2.167, 95% CI: 0.569–8.256).

Mammography was performed in 18 cases, showing no suspicious findings in two (11.1%), mass lesions in six (33.3%), and non-mass findings in 10 (55.5%). Pathological calcifications were detected in 13 cases (72.2%). Although individual mammographic findings did not reach statistical significance (*p* > 0.05), analysis of effect sizes revealed distinct clinical trends. Specifically, Group 2 exhibited higher odds of presenting with pathological calcifications (OR = 5.000, 95% CI: 0.540–46.284, *φ* = 0.338) and mammographic mass lesions (OR = 3.571, 95% CI: 0.320–39.878, *φ* = 0.247), both displaying moderate effect magnitudes compared with Group 1.

MRI was available for 11 lesions, of which 10 (90.9%) showed mass enhancement. Most lesions demonstrated type 3 (81.8%) kinetic enhancement curves. Satellite nodules were observed in four lesions (36.4%). No statistically significant differences were observed across individual MRI parameters (*p* > 0.05). However, effect size analysis revealed clinical trends despite the small cohort size. Mass-like enhancement predominated in both groups (*φ* = 0.289), with non-mass enhancement occurring in a single patient in Group 2. Regarding kinetic features, Group 1 demonstrated a higher frequency of Type 3 curves compared with Group 2 (100.0% vs. 66.6%), reflecting a medium-to-large effect size (*φ* = 0.404). Additionally, satellite nodules were less frequent in Group 1 than in Group 2 (20.0% vs. 50.0%), exhibiting a moderate effect size (*φ* = −0.306, OR = 4.000, 95%CI: 0.259–61.801).

### 3.3. Histopathologic Features

Of the 34 patients, in situ disease occurred in five (14.7%; Group 1: *n* = 3, Group 2: *n* = 2), while 29 had invasive tumors (85.3%; Group 1: *n* = 11, Group 2: *n* = 18). Although Group 1 exhibited a higher proportion of in situ tumors compared with Group 2 (21.4% vs. 10.0%; OR = 2.455, 95% CI: 0.352–17.112), this difference did not reach statistical significance (*p* = 0.380) and demonstrated a small-to-medium effect size (*φ* = 0.159). Most of the invasive tumors were grade II (51.7%) and III (44.8%). The distribution of tumor grades did not differ significantly between the two groups. ER and PR positivity rates were each 55.9% (19/34), while 76.4% (26/34) of tumors were HER2−negative. Immunohistochemical classification showed a predominance of luminal B (47.1%) and luminal A (23.5%) subtypes, followed by triple-negative (20.6%) and HER2+ (8.8%) diseases. No statistically significant differences were observed between groups regarding receptor status or molecular subtype distribution. Rates of ER positivity (*p* = 0.491, OR = 1.800, 95% CI: 0.435–7.447, *φ* = 0.141), PR positivity (*p* = 0.729, OR= 0.667, 95% CI: 0.165–2.688, *φ* = −0.098), and HER2 over-expression (*p* = 1.000, OR= 0.818, 95% CI: 0.158–4.225, *φ* = −0.042) were comparable across cohorts. Similarly, the mean Ki-67 proliferation index was slightly higher in Group 1, but this difference did not reach statistical significance (*p* = 0.540), demonstrating a small effect size (Cohen’s d = 0.219). Table 2 provides a comparison of detailed pathological tumor characteristics between the two age groups.

### 3.4. Treatment Protocols and Follow-Up

Treatment protocols were determined by our institutional multidisciplinary team. Among the patients included in the study, 13 underwent breast-conserving surgery (BCS) (Group 1: *n* = 7, 50%; Group 2: *n* = 6, 30%), 14 underwent mastectomy (Group 1: *n* = 6, 42.9%; Group 2: *n* = 8, 40%), and 7 received NCT (Group 1: *n* = 1, 7.1%; Group 2: *n* = 6, 30%) as part of their initial treatment approach. There was no statistically significant difference in the distribution of initial treatment modalities between Group 1 and Group 2 (*p* = 0.276). pTNM staging was assessed using surgical specimens; 7 patients who underwent NCT before surgery were excluded from this analysis. T1 and T2 were the most frequent tumor stages (25.9% each), while the majority of patients (63%) were node-negative (N0). Distant metastasis at the time of surgery was absent in 26 patients (M0), while one patient had metastatic disease (M1) (3.7%). According to the AJCC system, the most prevalent stages were II and III (29.6% each). There was no statistically significant difference in pathological stage distribution between Group 1 and Group 2. Furthermore, when patients were grouped into early stage (0–II) and advanced stage (III–IV) disease, no statistically significant difference was observed between the ≤30-year group (53.9% early vs. 46.1% advanced) and the 31–35-year group (78.6% early vs. 21.4% advanced) (*p* = 0.236).

**Figure 4:**
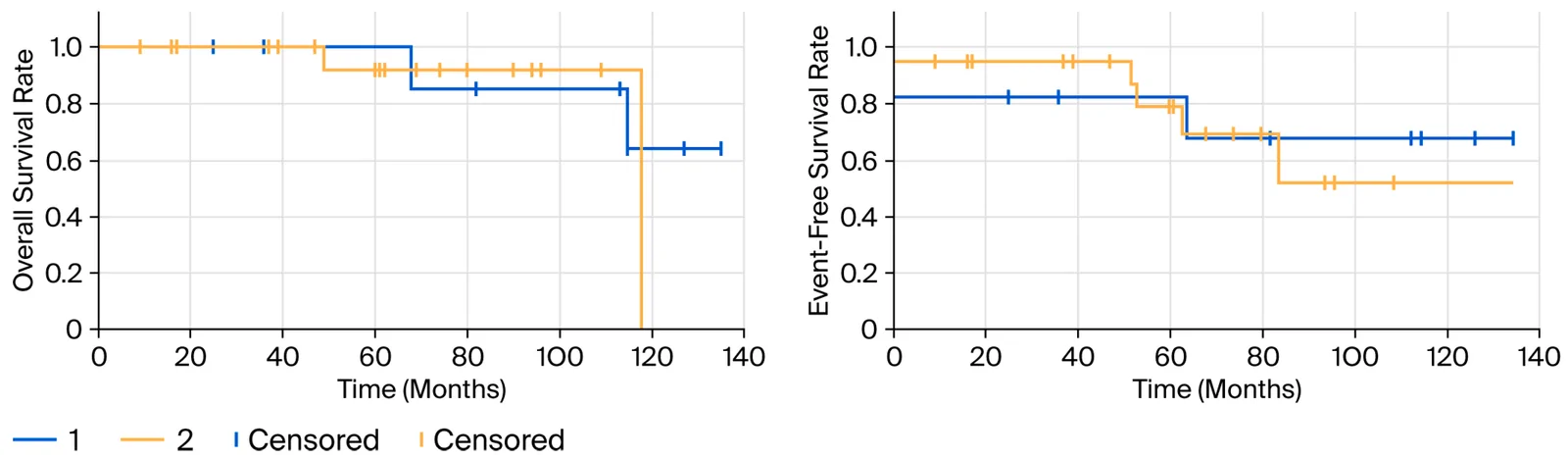
Kaplan–Meier survival curves comparing OS and EFS between Group 1 (≤30 years) and Group 2 (31–35 years) patients. No statistically significant differences were observed between the groups for OS (*p* = 0.784) or EFS (*p* = 0.897). EFS: event-free survival; OS: overall survival.

All patients had a minimum follow-up duration of six months. Survival analyses included OS and EFS. Follow-up data were available for 30 patients, consisting of 11 in Group 1 and 19 in Group 2. Four patients who had no follow-up visit within the past year were excluded from the analysis. The median OS time was 68 months (range: 9–135) for all patients, 82 months in Group 1, and 62 months in Group 2. The estimated OS rates were 100% in both groups at 1 year, 100% in Group 1 and 92.3% in Group 2 at 5 years. During the follow-up period, a total of four deaths (13.3%) occurred—two in Group 1 and two in Group 2. None of the evaluated variables—including age (*p* = 0.919), ER (*p* = 0.478), PR (*p* = 0.935), HER2 (*p* = 0.102), molecular subtype (*p* = 0.068), tumor grade (*p* = 0.309), and pTNM stage (*p* = 0.594)—showed a statistically significant association with OS. The median EFS was 60.5 months (range: 6–135) for all patients, 64 months in Group 1, and 60 months in Group 2. Metastatic events occurred in three patients (27.3%) in Group 1 and five patients (26.3%) in Group 2. The estimated EFS rates were 81.8% in Group 1 and 94.7% in Group 2 at 1 year; 81.8% in Group 1 and 84.2% in Group 2 at 5 years (Table 3). Kaplan–Meier analysis demonstrated that the difference in OS and EFS distributions was not statistically significant between the two age groups (*p* = 0.784 and *p* = 0.897, respectively) (Figure 4).

## 4. Discussion

This 14-year retrospective study of BC in women aged ≤35 years showed no significant differences in clinicopathological features, imaging findings, or long-term survival between the ≤30 and 31–35 age groups. Although advanced-stage disease was more frequent in the younger cohort, this was not statistically significant. The co-occurrence of more advanced-stage disease with favorable survival in this cohort is very likely attributable to the small sample size and limited event number rather than a true biological association and should not be over-interpreted. Both OS and EFS were favorable, and none of the assessed variables, including age itself, showed a significant association with OS.

Definitions of “young age” BC are inconsistent in the literature, with various thresholds across clinical guidelines. The European School of Oncology (ESO) and the European Society of Medical Oncology (ESMO) International Consensus Guidelines, along with numerous recent studies, use an age threshold of under 40 years. However, there is a growing acknowledgment of a distinct subgroup referred to as “very young,” usually defined as 35 years or younger [18,24,25,26]. In the present study, we focused specifically on patients aged ≤ 35 years to better reflect the epidemiological landscape in our country, where the median age of BC diagnosis is approximately 51 years, nearly a decade earlier than in high-income Western populations [19,20,27,28]. This recalibration minimizes the influence of the 36–40 age range, which in our region represents a more common premenopausal demographic rather than an early-onset group, and provides clearer insights into the unique aggressive features of truly young patients. Our approach is further supported by previous research indicating that the prognostic impact of young age is most pronounced for those under 35, where risks of recurrence and mortality increase more dramatically compared with the 36–40 age group [29,30].

Detecting BC in young women is challenging due to high breast density, and concerns regarding cumulative radiation exposure, which limit mammographic evaluation. Consequently, US is the preferred initial imaging technique for diagnostic evaluation. In our cohort, breast density was predominantly high (BI-RADS C/D) as expected, and US identified masses in all patients, which corresponded to palpable lumps in most cases, highlighting the utility of targeted US in enhancing cancer detection in young women. This aligns with previous reports where masses were the primary US finding (95.7%) in women < 30 years, compared to rare intraductal calcifications [31]. Mammography showed an 88.9% positive predictive value, consistent with the 50–90% range reported in the literature [31,32,33]. While no suspicious findings were observed in 2 cases (11.1%), pathological calcifications were present in 72.2%, underscoring their importance as a reliable diagnostic clue. In contrast to our series, where no patient was diagnosed solely based on mammographic microcalcifications, Durhan et al. reported a small subset (3.8%) in whom US was normal and the diagnosis was established through microcalcifications [34]. Breast MRI was primarily utilized to assess tumor extent in patients being considered for BCS, of which 10 (90.9%) showed mass enhancement. Most lesions demonstrated type 2 or type 3 kinetic enhancement curves, consistent with previously published data [31,32,33,35].

In our cohort, invasive ductal carcinoma (IDC) was the most common histological subtype (79.5%), followed by ductal carcinoma in situ (DCIS) (14.7%), similar to reports by An et al. (<30 years BC; IDC: 72%, DCIS: 12%) [31]. The most frequent molecular subtype was luminal B (47.1%), followed by luminal A (23.5%), consistent with a predominance of hormone receptor-positive disease (ER+: 55.9%) in our cohort. Notably, histological and molecular subtypes did not differ significantly between our age subgroups. This contrasts with studies comparing young to older women; for instance, Colleoni et al. found higher rates of ER− (*p* < 0.001) and PR− (*p* = 0.001) tumors in patients < 35 compared with those 35–50 years [15]. Similarly, Kheirelseid et al. reported increased ER negativity (*p* < 0.001) and HER2 positivity (*p* = 0.002) in patients <40 compared with those >40 [36]. These findings suggest that while biological differences are evident when comparing young to older BC patients, such heterogeneity may not be apparent within the already young population of women aged ≤ 35 years.

This study showed no significant differences between age groups regarding T stage, nodal involvement, or AJCC staging. Although T1 tumors were less common and T4 tumors were more frequent in patients ≤ 30 years, this did not reach statistical significance. Similarly, prior studies reported no significant T-stage differences when comparing patients < 35 and 35–50 years [6,15]. Overall, 63% of our cohort was N0, consistent with An et al., who reported 42% nodal positivity in patients <30 years [31]. Additionally, another study showed no clear trends in nodal involvement across various young age groups (<35, 35–40, 41–45, and 46–50 years) [15], suggesting that nodal burden may not differ by age within this population. Consistent with previous reports [11,14], advanced disease (AJCC stages III–IV) was more frequent in our ≤30 group, though this difference was not statistically significant, which may have been precluded by our limited sample size. These findings collectively suggest that while very young patients often present with advanced disease, significant staging differences may not exist within the ≤35-year demographic.

In our cohort, OS and EFS were favorable, with no significant differences detected between age groups. In this exploratory analysis, none of the evaluated clinicopathological factors, including age itself, was significantly associated with OS or EFS; given the small sample size and limited number of events, these findings should be regarded as hypothesis-generating rather than confirmatory. Prior studies have reported conflicting results. While some studies report worse OS in patients < 35 years [6], others suggest age is not an independent prognostic factor after adjusting for tumor size, stage, and receptor status [36]. Similarly, while certain reports show higher recurrence rates in younger women [6,37,38,39], others found no disease-free survival differences [36,39,40]. These discrepancies may be explained by several limitations, including age thresholds, a small number of patients, varying study periods, insufficient pathological or molecular data, and heterogeneous case populations in terms of ethnicity and treatment approaches. By focusing exclusively on the ≤35-year demographic, our study indicates that age itself may not be the dominant determinant of outcome within this specific subgroup. Furthermore, although a limited sample size precluded molecular subgroup analysis, previous research highlights the need to clarify how BC subtypes modify age-related prognosis [14,41,42,43].

Our study has several limitations, primarily its retrospective single-center design and small sample size, which may constrain the generalizability of the findings. The low number of survival events precluded multivariable Cox regression analysis, potentially limiting the identification of independent prognostic factors. Additionally, the long time span covered by our study also raises the issue of evolving treatment approaches: in recent years, breast cancer treatment has become significantly less invasive, with breast-conserving therapy remaining the most common surgical approach while axillary dissection has declined markedly, and neoadjuvant systemic therapy—including endocrine therapy in ER+/HER2− disease—has become increasingly standard, particularly for triple-negative and HER2+ disease [44]. As our 14-year study period (2011–2024) spans this evolution, treatment-related heterogeneity across the cohort is a relevant limitation not fully captured by describing management as following “the same institutional protocols.” In our cohort, 7 of 34 patients (20.6%) received NCT, a proportion consistent with the broader shift toward neoadjuvant approaches described above, though our retrospective design did not allow us to assess whether NCT selection criteria or systemic regimens changed across the 14-year period. Treatment decisions were nonetheless guided by the same institutional multidisciplinary team throughout, though the cohort size prevented formal comparisons between different treatment modalities. As another limitation, although tumor pathology was comprehensively assessed, genetic testing could not be performed in all patients. Finally, although the median follow-up was 68 months, the minimum six-month follow-up for some patients may be considered short for long-term survival analysis.

Despite these limitations, the present study offers several notable strengths. First, to our knowledge, this is one of the few studies in the literature to directly compare two subgroups within the young BC population, those aged 30 years or younger and those aged 31–35 years, rather than benchmarking against postmenopausal or older premenopausal counterparts, thereby providing a more refined analysis of age-related prognostic differences within this demographic. Second, all patients were managed according to the same institutional multidisciplinary protocols throughout the 14-year study period, minimizing treatment-related heterogeneity that commonly confounds multi-center retrospective analyses. Third, imaging findings were systematically reviewed by two radiologists, one of whom was a breast radiologist with over two decades of expertise, with all discrepancies resolved by consensus, lending a high degree of reproducibility to the radiological findings. Fourth, the study integrates imaging characteristics, detailed pathological variables including immunohistochemical profiling, and long-term survival data, offering a comprehensive and multidimensional perspective on young-onset BC.

In conclusion, the findings of the present study indicate that, within the studied population aged 35 and under, being aged 30 or younger is not associated with worse outcomes compared with being in the 31–35 age range. The present work may be regarded as a preliminary report. Future research conducted in multi-center cohorts with extended follow-up periods is warranted to further elucidate long-term survival outcomes and recurrence patterns in this population with a long life expectancy.

## Figures and Tables

**Table 1:** Imaging findings of patients.

Patients	Group 1		Group 2		*p*-Value	Effect Size	All	
	*n* = 14	%	*n* = 20	%			*n* = 34	%
Breast density (ACR BI-RADS)								
A	2	14.3%	0	0%	0.027 *	0.473	2	5.9%
B	1	7.1%	0	0%			1	2.9%
C	6	42.9%	17	85%			23	67.6%
D	5	35.7%	3	15%			8	23.6%
Distribution								
Unifocal	9	64.3%	13	65%	0.827	0.126	22	64.7%
Multifocal	5	35.7%	6	30%			11	32.4%
Multicentric	0	0%	1	5%			1	2.9%
Lesions **	*n* = 15	%	*n* = 25	%			*n* = 40	%
Ultrasound	15	100%	25	100%			40	100%
Lesion					0.435	0.128		
Non-mass	4	36.4%	4	16%			8	20%
Mass	11	63.6%	21	84%			32	80%
Shape					0.584	0.125		
Round/oval	2	18.2%	2	9.5%			4	12.5%
Irregular	9	81.8%	19	90.5%			28	87.5%
Margins					0.063	0.470		
Circumscribed	0	0%	2	9.5%			2	6.3%
Indistinct	2	18.2%	3	14.3%			5	15.6%
Microlobulated	2	18.2%	11	52.4%			13	40.6%
Spiculated	7	63.6%	5	23.8%			12	37.5%
Echo pattern					0.781	0.102		
Hypoechoic	8	72.7%	17	80.9%			25	78.1%
Heterogeneous	2	18.2%	3	14.3%			5	15.6%
Complex	1	9.1%	1	4.8%			2	6.3%
Calcifications					0.231	0.310		
None	4	36.4%	12	57.1%			16	50%
Within mass	7	63.6%	7	33.3%			14	43.7%
Outside mass	0	0%	2	9.6%			2	6.3%
Associated features								
Distortion	3	20%	5	20%	1.000	0.000	8	20%
Duct changes	3	20%	3	12%	0.661	0.111	6	15%
Skin retraction	0	0%	1	4%	1.000	−0.124	1	2.5%
Skin thickening	2	13.3%	1	4%	0.548	0.170	3	7.5%
Edema	0	0%	1	4%	1.000	−0.124	1	2.5%
BI-RADS					0.320	0.181		
4	5	33.3%	13	52%			18	45%
5	10	66.6%	12	48%			22	55%
Mammography	6	40%	12	60%			18	45%
No suspicious findings	2	33.3%	0	0%	0.098	−0.516	2	11.1%
Mass	1	16.6%	5	41.7%	0.600	0.247	6	33.3%
Non-mass	3	50%	7	58.3%	1.000	0.079	10	55.5%
Pathological calcifications	3	50%	10	83.3%	0.248	0.338	13	72.2%
MRI	5	33.3%	6	30%			11	27.5%
Enhancement pattern					1.000	0.289		
Mass	5	100%	5	83.3%			10	90.9%
Non-mass	0	0%	1	16.7%			1	9.1%
Kinetic enhancement curve					0.455	0.404		
Type 2	0	0%	2	33.3%			2	18.2%
Type 3	5	100%	4	66.6%			9	81.8%
Satellite nodules	1	20%	3	50%	0.545	−0.306	4	36.4%

* *p*-value < 0.05 indicates statistical significance (non-significant *p*-values should not be interpreted as evidence of equivalence between groups). ** Imaging findings were analyzed on a per-lesion basis. ACR BI-RADS: American College of Radiology Breast Imaging Reporting and Data System; MRI: magnetic resonance imaging.

**Table 2:** Pathological tumor data.

	Group 1		Group 2		*p*-Value	Effect Size	All	
	*n* = 14	%	*n* = 20	%			*n* = 34	%
Histologic Tumor Type					0.662	0.245		
DCIS	3	21.4%	2	10%			5	14.7%
LCIS component	1	7.1%	0	0%			1	2.9%
IDC	10	71.5%	17	85%			27	79.5%
DCIS component	8	57.1%	8	40%			16	47.1%
Invasive metaplastic carcinoma	1	7.1%	0	0%			1	2.9%
Invasive mixed papillary-ductal carcinoma	0	0%	1	5%			1	2.9%
Tumor category					0.380	0.159		
In situ tumors	3	21.4%	2	10%			5	14.7%
Invasive tumors	11	78.6%	18	90%			29	85.3%
Receptor status								
ER+	9	64.3%	10	50%	0.491	0.141	19	55.9%
PR+	7	50%	12	60%	0.729	−0.098	19	55.9%
HER2+	3	21.4%	5	25%	1.000	−0.042	8	23.6%
Ki-67 **	38 ± 16		33 ± 27		0.540	0.219	35 ± 24	
Molecular subtypes					0.771	0.203		
Luminal A	2	14.3%	6	30%			8	23.6%
Luminal B	8	57.2%	8	40%			16	47%
HER2-enriched	1	7.1%	2	10%			3	8.8%
Triple negative	3	21.4%	4	20%			7	20.6%

*p*-value < 0.05 indicates statistical significance (non-significant *p*-values should not be interpreted as evidence of equivalence between groups). ** Mean ± SD. DCIS: ductal carcinoma in situ; ER: estrogen receptor; HER2: human epidermal growth factor receptor 2; IDC: invasive ductal carcinoma; LCIS: lobular carcinoma in situ; PR: progesterone receptor.

**Table 3:** Survival outcomes.

	Group 1	Group 2	*p*-Value
Included in survival analysis, *n*	11	19	—
Deaths, *n* (%)	2 (18.2%)	2 (10.5%)	—
Recurrence/metastasis, *n* (%)	3 (27.3%)	5 (26.3%)	—
OS (median)	82	62	0.784 *
1-year OS	100%	100%	
5-year OS	100%	92.3%	
EFS (median)	64	60	0.897 *
1-year EFS	81.8%	94.7%	
5-year EFS	81.8%	84.2%	

* Calculated using the Log-rank test comparing Group 1 and Group 2. EFS: event-free survival; OS: overall survival.

## Data Availability

The data presented in this study are available from the corresponding author upon reasonable request. The data are not publicly available due to patient privacy and ethical restrictions.

## Supplementary Materials

The following supporting information can be downloaded at: https://www.mdpi.com/article/10.3390/diagnostics16162510/s1, Table S1: Detailed imaging findings and pathological tumor data.

## Abbreviations

The following abbreviations are used in this manuscript:

ACR	American College of Radiology
BC	Breast cancer
BI-RADS	Breast Imaging Reporting and Data System
CC	Craniocaudal
DCIS	Ductal carcinoma in situ
EFS	Event-free survival
ER	Estrogen receptor
ESMO	European Society for Medical Oncology
ESO	European School of Oncology
FISH	Fluorescence in situ hybridization
FNAB	Fine-needle aspiration biopsy
HER2	Human epidermal growth factor receptor 2
IDC	Invasive ductal carcinoma
ILC	Invasive lobular carcinoma
IQR	Interquartile range
LCIS	Lobular carcinoma in situ
LIQ	Lower inner quadrant
LOQ	Lower outer quadrant
LVI	Lymphovascular invasion
MIP	Maximum intensity projection
MLO	Mediolateral oblique
MRI	Magnetic resonance imaging
NCT	Neoadjuvant chemotherapy
OS	Overall survival
PR	Progesterone receptor
SD	Standard deviation
TNM	Tumor-Node-Metastasis
UIQ	Upper inner quadrant
UOQ	Upper outer quadrant
US	Ultrasound
