# Supplementary material for: Breast Cancer in Women Aged ≤35 Years: A Single-Center Retrospective Comparative Analysis by Age Subgroup
Source: Diagnostics (Basel). 2026 Aug 9;16(16):2510. doi: 10.3390/diagnostics16162510 (PMC13511427; doi:10.3390/diagnostics16162510)
Supplement: Supplementary file 1 [file diagnostics-16-02510-s001.zip › diagnostics-4378844-supplementary.pdf]

Supplementary Table S1. Detailed imaging findings and pathological tumor data

* *p*-value <0.05 indicates statistical significance (non-significant *p*-values should not be interpreted as evidence of equivalence between groups)

[1] For additional analysis, lesions located in the upper and/or lower half of the breast were counted in both relevant quadrant categories

[2] Imaging findings were analyzed on a per-lesion basis

** Mean ± SD

*** pTNM and LVI was assessed using surgical specimens and included 27 patients; 7 patients who underwent NCT before surgery were excluded from this analysis

ACR BI-RADS: American College of Radiology Breast Imaging Reporting and Data System; AJCC: American Joint Committee on Cancer; DCIS: ductal carcinoma in-situ; ER: estrogen receptor; HER2: human epidermal growth factor receptor 2; IDC: invasive ductal carcinoma; ILC: invasive lobular carcinoma; LCIS: lobular carcinoma in-situ; LIQ: lower-inner quadrant; LOQ: lower-outer quadrant; LVI: lymphovascular invasion; UIQ: upper-inner quadrant; UOQ: upper-outer quadrant; MRI: magnetic resonance imaging; NCT; neoadjuvant chemotherapy; PR: progesterone receptor; pTNM: pathological tumor-node-metastasis

**Patients**	**Group 1**		**Group 2**		***p*-value**	**Effect size**	**All**	
	***n* = 14**	**%**	***n* = 20**	**%**			***n* = 34**	**%**
Breast density (ACR BI-RADS)								
A	2	14.3%	0	0%	0.027*	0.473	2	5.9%
B	1	7.1%	0	0%			1	2.9%
C	6	42.9%	17	85%			23	67.6%
D	5	35.7%	3	15%			8	23.6%
Laterality								
Unilateral	14	100%	20	100%	0.291	0.214	34	100%
Right	10	71.4%	10	50%			20	58.8%
Left	4	28.6%	10	50%			14	41.2%
Bilateral	0	0%	0	0%			0	0%
Location[1]								
UOQ	9	56.2%	15	65.3%	0.627	0.211	24	61.5%
LUQ	4	25%	3	13%			7	17.9%
LIQ	0	0%	2	8.7%			2	5.1%
UIQ	3	18.8%	3	13%			6	15.4%
Distribution								
Unifocal	9	64.3%	13	65%	0.827	0.126	22	64.7%
Multifocal	5	35.7%	6	30%			11	32.4%
Multicentric	0	0%	1	5%			1	2.9%
Lesions[2]	*n* = 15	%	*n* = 25	%			*n* = 40	%
Ultrasound	15	100%	25	100%			40	100%
Lesion					0.435	0.128		
Non-mass	4	36.4%	4	16%			8	20%
Mass	11	63.6%	21	84%			32	80%
Shape					0.584	0.125		
Round/oval	2	18.2%	2	9.5%			4	12.5%
Irregular	9	81.8%	19	90.5%			28	87.5%
Margins					0.063	0.470		

Circumscribed	0	0%	2	9.5%			2	6.3%
Indistinct	2	18.2%	3	14.3%			5	15.6%
Microlobulated	2	18.2%	11	52.4%			13	40.6%
Spiculated	7	63.6%	5	23.8%			12	37.5%
Echo pattern					0.781	0.102		
Hypoechoic	8	72.7%	17	80.9%			25	78.1%
Heterogeneous	2	18.2%	3	14.3%			5	15.6%
Complex	1	9.1%	1	4.8%			2	6.3%
Calcifications					0.231	0.310		
None	4	36.4%	12	57.1%			16	50%
Inmass	7	63.6%	7	33.3%			14	43.7%
Outside mass	0	0%	2	9.6%			2	6.3%
Associated features								
Distortion	3	20%	5	20%	1.000	0.000	8	20%
Duct changes	3	20%	3	12%	0.661	0.111	6	15%
Skin retraction	0	0%	1	4%	1.000	-0.124	1	2.5%
Skin thickening	2	13.3%	1	4%	0.548	0.170	3	7.5%
Edema	0	0%	1	4%	1.000	-0.124	1	2.5%
BI-RADS					0.320	0.181		
4	5	33.3%	13	52%			18	45%
5	10	66.6%	12	48%			22	55%
Mammography	6	40%	12	60%			18	45%
No suspicious findings	2	33.3%	0	0%	0.098	-0.516	2	11.1%
Mass	1	16.6%	5	41.7%	0.600	0.247	6	33.3%
Non-mass	3	50%	7	58.3%	1.000	0.079	10	55.5%
Pathological calcifications	3	50%	10	83.3%	0.248	0.338	13	72.2%
MRI	5	33.3%	6	30%			11	27.5%
Enhancement pattern					1.000	0.289		
Mass	5	100%	5	83.3%			10	90.9%
Non-mass	0	0%	1	16.7%			1	9.1%
Kinetic enhancement curve					0.455	0.404		
Type 2	0	0%	2	33.3%			2	18.2%
Type 3	5	100%	4	66.6%			9	81.8%
Satellite nodules	1	20%	3	50%	0.545	-0.306	4	36.4%
Histologic tumor type					0.662	0.245		
DCIS	3	21.4%	2	10%			5	14.7%
LCIS component	1	7.1%	0	0%			1	2.9%
IDC	10	71.5%	17	85%			27	79.5%
DCIS component	8	57.1%	8	40%			16	47.1%
Invasive metaplastic carcinoma	1	7.1%	0	0%			1	2.9%
Invasive mixed papillary-ductal carcinoma	0	0%	1	5%			1	2.9%
Tumor category					0.380	0.159		
In-situ tumors	3	21.4%	2	10%			5	14.7%
Invasive tumors	11	78.6%	18	90%			29	85.3%

Grade								
In-situ tumors					0.283	0.334		
I	0	0%	1	5%			1	2.9%
II	1	7.1%	1	5%			2	5.9%
III	2	14.3%	0	0%			2	5.9%
Invasive tumors					0.584	0.201		
I	1	7.1%	0	0%			1	2.9%
II	5	35.7%	10	50%			15	44.1%
III	5	35.7%	8	40%			13	38.3%
Receptor status								
ER +	9	64.3%	10	50%	0.491	0.141	19	55.9%
PR +	7	50%	12	60%	0.729	-0.098	19	55.9%
HER2 +	3	21.4%	5	25%	1.000	-0.042	8	23.6%
Ki-67**	38±16		33±27		0.540	0.219	35±24	
Molecular subtypes					0.771	0.203		
Luminal A	2	14.3%	6	30%			8	23.6%
Luminal B	8	57.2%	8	40%			16	47%
HER2 enriched	1	7.1%	2	10%			3	8.8%
Triple negative	3	21.4%	4	20%			7	20.6%
pTNM Stage (AJCC 8th Edition)**								
T					0.283	0.404		
Tis	3	23.1%	2	14.3%			5	18.5%
1	1	7.7%	6	42.8%			7	25.9%
2	5	38.4%	2	14.3%			7	25.9%
3	0	0%	2	14.3%			2	7.4%
4	4	30.8%	2	14.3%			6	22.2%
N					1.000	0.030		
0	8	61.5%	9	64.3%			17	63%
1	2	15.4%	2	14.3%			4	14.8%
2	2	15.4%	2	14.3%			4	14.8%
3	1	7.7%	1	7.1%			2	7.4%
M					0.481	0.203		
0	12	92.3%	14	100%			26	96.3%
1	1	7.7%	0	0%			1	3.7%
Stage					0.589	0.334		
0	3	23.1%	2	14.3%			5	18.5%
1	1	7.7%	4	28.6%			5	18.5%
2	3	23.1%	5	35.7%			8	29.6%
3	5	38.4%	3	21.4%			8	29.6%
4	1	7.7%	0	0%			1	3.7%
LVI***	6	46.1%	5	35.7%	0.704	0.105	11	40.7%
